# Diagnostic challenges in a diffuse large B-cell lymphoma of the maxilla presenting as exposed necrotic bone

**DOI:** 10.4317/jced.59346

**Published:** 2022-03-01

**Authors:** Emmanouil Vardas, Maria Georgaki, Erofili Papadopoulou, Konstantina Delli, Andreas Kouroumalis, Evangelos Kalfarentzos, Eleftheria Lakiotaki, Nikolaos G. Nikitakis

**Affiliations:** 1 DDS, MSc, PhD, Department of Oral Medicine & Pathology and Hospital Dentistry, School of Dentistry, National and Kapodistrian University of Athens, Greece; 2 DDS, Dr med dent, PhD, Department of Oral Diseases - Oral and Maxillofacial Surgery, University of Groningen, University Medical Center Groningen, Netherlands; 3 MD, DDS, Department of Oral and Maxillofacial Surgery, School of Dentistry, National and Kapodistrian University of Athens, Greece; 4 MD, DDS, PhD, Department of Oral and Maxillofacial Surgery, School of Dentistry, National and Kapodistrian University of Athens, Greece; 5 MD, PhD, First Department of Pathology, School of Medicine, National and Kapodistrian University of Athens, Greece; 6 MD, DDS, PhD, Department of Oral Medicine & Pathology and Hospital Dentistry, School of Dentistry, National and Kapodistrian University of Athens, Greece

## Abstract

Lymphoma is the second most common malignancy in the head and neck area, affecting both nodal and extranodal sites, including oral soft and hard tissues, usually in the form of non-Hodgkin’s lymphoma (NHL). However, lymphomas of the jaws, including diffuse large B-cell lymphoma (DLBCL), the most common type of NHL, are very rare and may cause significant diagnostic challenges resembling common jaw pathologies, such as periapical lesions, osteomyelitis and osteonecrosis. The aim of this paper is to present a rare case of DLBCL in an 84-years-old diabetic male patient on methylprednisolone treatment for autoimmune hemolytic anemia. The lesion appeared clinically as exposed necrotic bone of the maxilla with surrounding soft tissue ulceration and radiographically as an extensive osteolytic lesion with ill-defined borders. Despite the resemblance of the lesion with osteonecrosis or osteomyelitis that could be theoretically related to diabetes and/or systemic use of corticosteroids, histopathologic examination, necessitating a repeat biopsy in order to acquire sufficient tissue, revealed the final diagnosis of lymphoma. The need for increased clinical awareness and vigilance of this possible diagnostic conundrum is emphasized.

** Key words:**Diffuse large B-cell lymphoma, exposed bone, oral, malignancy, maxilla, jaw osteonecrosis, differential diagnosis.

## Introduction

Lymphoma is a group of lymphoreticular malignancies, classically divided in Hodgkin’s lymphoma (HL) and Non-Hodgkin’s lymphoma (NHL) (1). HL accounts for 10% of all lymphomas, often presenting as nodal disease, commonly involving cervical, axillary and inguinal nodes (1,2). NHL corresponds to the vast majority (90%) of lymphomas and also frequently affects lymph nodes, but, in up to 40% of cases, it involves extranodal sites, most commonly the gastrointestinal tract, skin, bone and brain (3). If tonsils and Waldeyer’s ring are included, head and neck represents the second most frequently affected site by extranodal NHL, following gastrointestinal tract (3); vice versa, NHL is the second most common type of cancer in the head and neck area (4), accounting for approximately 5% of all malignancies in this anatomic area (5,6). Oral cavity is a relatively rare site for extranodal NHLs, representing approximately 3-5% of all lymphomas; on the other hand, lymphomas account for approximately 3.5% of all intraoral malignancies (4,7-15). Oral NHL usually affects patients older than 50 years of age and may develop as primary disease or be part of a disseminated process (4,12). Both soft tissues (such as palatal mucosa, gingiva, buccal mucosa, tongue, and floor of mouth) and jaw bones can be affected, the latter accounting for approximately one third to one half of all oral cases (4,7-17). Although some authors have suggested that oral intraosseous lymphomas may have a worse prognosis compared to their soft tissue counterparts due to a more advanced clinical stage at diagnosis (7), others have not found significant differences in survival time between these two groups (10).

Diffuse large-B cell-lymphoma (DLBCL) is the most common type of NHL, including the head and neck area (1,18,19). It is an aggressive neoplasm of medium or large lymphoid cells, clinically appearing as a rapidly growing mass, and commonly occurs in men older than 50 years (18). Although DLBCL appears to be the most common type of NHL in the oral cavity (e.g. 50%, 58% and 68% of cases in the series of van der Waal *et al*. (11), Kemp *et al*. (12) and Solomides *et al*. (9), respectively), relatively few publications have focused on this entity and its diagnosis remains challenging, especially considering the variety of its clinical presentations (20-32).

The aim of the present paper is to describe a rare case of DLBCL affecting the maxilla, presenting as exposed necrotic bone and resembling osteomyelitis or osteonecrosis of the jaws (ONJ) in order to highlight the possibility of such an unusual clinical appearance and to increase awareness of the possible occurrence of this entity in the oral cavity.

## Case Report

An 84 years old male patient presented to the Department of Oral Medicine & Pathology and Hospital Dentistry, with a chief complaint of exposed bone in the maxilla of 15 days duration and loss of a tooth in the same area, 5 days prior to the initial evaluation. Patient’s medical history was significant for diabetes mellitus (DM) type II, as well as autoimmune hemolytic anemia (managed with long-term methylprednisolone administration), hypertension, benign prostate hyperplasia, hyperuricemia and hypothyroidism, all under medications. There was no history of treatment with antiresorptive or antiangiogenic agents, nor previous radiation therapy in the head and neck region. A recent blood test was within normal limits.

**Figure 1:**
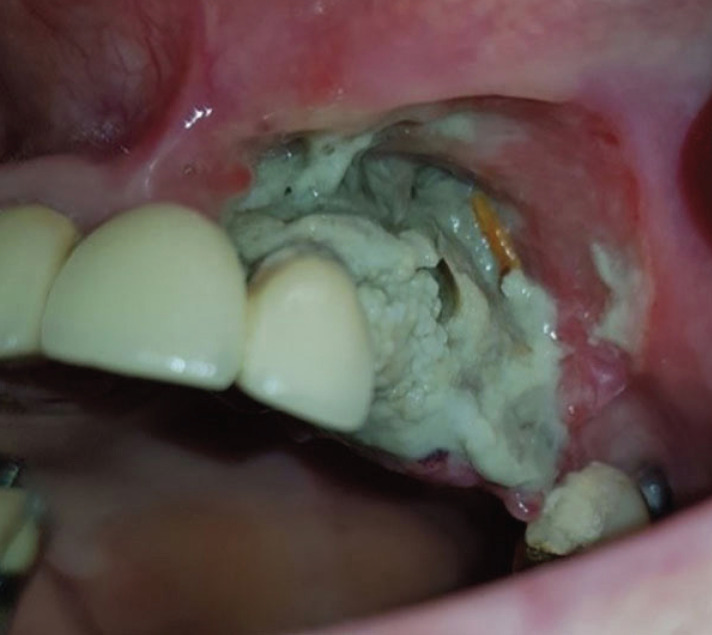
Clinical examination. Exposed bone in the left maxilla, partially covered by a greenish pseudomembrane with surrounding soft tissue ulceration.

**Figure 2:**
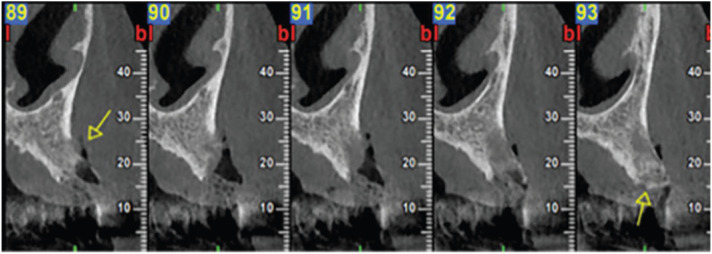
Imaging examination. Cone beam computed tomography scan showing osteolytic lesion with ill-defined borders and buccal and palatal perforation in the left maxillary alveolar bone between central incisor and second premolar.

**Figure 3:**
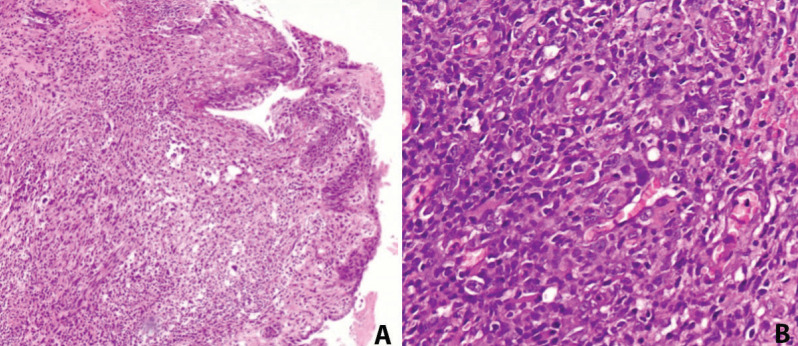
(A,B): Histopathologic examination (original magnification [A] ×100 and [B] ×400). Hematoxylin and eosin staining showing diffuse and dense infiltration of the connective tissue by large neoplastic lymphoid cells with anaplastic features, prominent nucleolus, and abundant cytoplasm.

**Figure 4:**
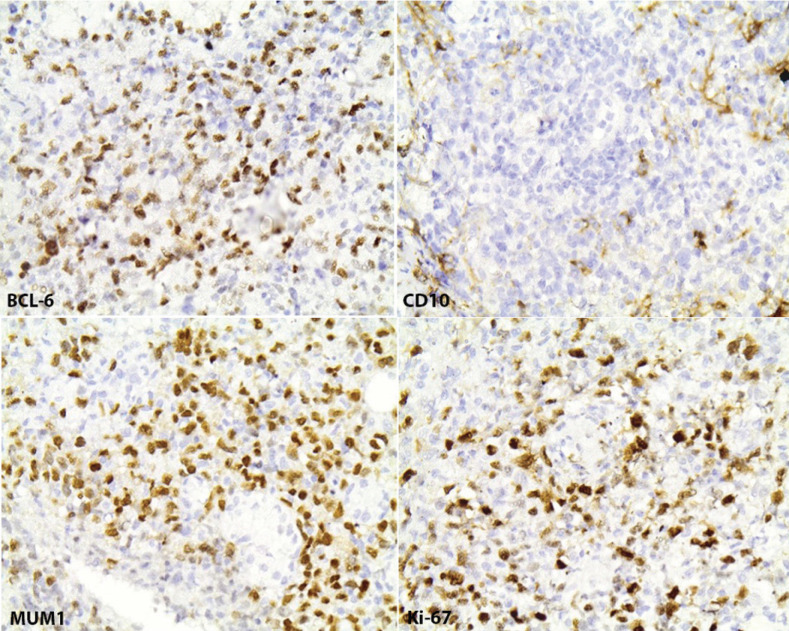
Immunohistochemical analysis (magnification x400) demonstrating diffuse and intense positivity of tumor cells for BCL-6 and MUM1 and a high Ki-67 index; CD10 was negative.

Clinical intraoral examination revealed exposed necrotic bone, partially covered by a greenish pseudomembrane with surrounding soft tissue ulceration, in the left maxilla, extending from the central incisor, approximately near superior labial frenum, to the second premolar, involving the free and attached gingiva and extending apically to the vestibular sulcus (Fig. 1). Cone beam computed tomography revealed an extensive osteolytic lesion with ill-defined borders and buccal and palatal cortical perforation in the left maxillary alveolar bone between central incisor and second premolar (Fig. 2). An initial incisional biopsy of the lesion was performed, and microscopic examination revealed only necrotic tissue. A complementary biopsy, extending into deeper and peripheral tissues, showed focal ulceration of the epithelium and diffuse infiltration of the connective tissue by large neoplastic lymphoid cells with anaplastic features and high mitotic index; extensive areas of tumor necrosis were also seen (Fig. 3a,b). Immunohistochemical evaluation showed positivity of the neoplastic cells for CD20, CD79a, MUM-1, CD30 and bcl-6, while CD3, CD10, CD5, CD4, CD21, CD15, IgA and EMA were negative. (Fig. 4) The tumor cell proliferation marker Ki-67 was highly expressed (90%) in the neoplastic cell population (Fig. 4). A final diagnosis of DLBCL, not otherwise specified (DLBCL-NOS), with a non-germinal center (non-GC) cell phenotype was established. The patient was referred to a hematology-oncology clinic for further evaluation and management. Unfortunately, the patient died of disease two months later.

## Discussion

NHL in the head and neck is relatively frequent, representing the second most common type of malignancy arising in this anatomical area, following squamous cell carcinoma (SCC) (4-6). Most of them are classified as of B cell origin, DLBCL being the most frequent subtype followed by mucosa-associated lymphoid tissue (MALT) lymphoma (4-6). Oral involvement by NHL has been reported to account for 3-5% of all lymphomas, DLBCL again representing the most common subtype (8,9,10,20). The etiology of primary DLBCLs of the oral cavity remains unknown (10,20), although an association with HIV infection has been noticed in a subset of patients (29). Based on a systematic review of 122 cases of oral DLBCL by Rodrigues-Fernandes *et al*. (29), the mean age of patients was 58.7 years, showing a male predilection (male to female ratio of approximately 3:2). The most commonly affected location was the gingiva (27%), followed by the mucosa of the maxilla, mandible and palate (25.4%, 15.6% and 14%, respectively). Clinically, they usually presented as a rapidly enlarging swelling with or without ulceration (20,21,29). The chief complaint of oral DLBCL patients is pain, followed by local numbness; other local symptoms include tooth mobility, dysphagia and nasal obstruction, while B symptoms are infrequently reported (11,25,29).

Radiographically, lymphoma involving bone typically presents as a poorly defined radiolucency (26,33). In a retrospective study, Mulligan *et al*. (33) described the imaging appearance of 237 cases of histopathologically-proven primary lymphoma of bone at various skeletal sites and showed that the majority of lesions presented as radiolucencies (70%), followed by a mixed appearance (28%); other common radiographic features included a moth-eaten pattern, periosteal reaction and presence of sequestra. Similarly, oral lymphomas radiographically demonstrate diffuse bone destruction with poorly defined borders, presenting as a solitary radiolucent lesion, causing loss of lamina dura and widening of the periodontal ligament or resembling common lesions, such as chronic apical periodontitis, periodontitis or osteomyelitis (11,15). Similar imaging features are seen in oral DLBCL (23-25), although atypical patters, such as generalized sclerosis of the maxilla, may be encountered (26).

Because of the non-pathognomonic and variable clinical and imaging features of oral DLBCL, its differential diagnosis is broad, ranging from inflammatory conditions of odontogenic or periodontal origin to several malignant neoplasms, including SCC, multiple myeloma, leukemia, osteosarcoma, and metastatic cancer (25). Disturbingly, due to overlapping characteristics, oral DLBCL can be misdiagnosed as similarly looking common pathologies, e.g. chronic periapical periodontitis and toothache (23,31). For example, in the case presented by Jessri *et al*. (23), a 32 years old patient with a swelling in the posterior mandible was initially managed with endodontic treatment for a perceived chronic periapical periodontitis of the second lower molar, before further imaging, biopsy and histopathologic examination revealed the malignant nature of the lesion. Obviously, misdiagnosis of a malignant lymphoma as a disease of odontogenic or periodontal origin may result not only in unnecessary dental interventions, but also in significant diagnostic and treatment delays (10).

The clinical and radiographic features of oral DLBCL may also simulate inflammatory jaw lesions, such as chronic osteomyelitis and osteonecrosis (22,27). In the present case, the clinical appearance of exposed bone and surrounding soft tissue ulceration along with the imaging features of osteolysis with ill-defined borders closely simulated osteonecrosis of the maxilla. However, there was no history of antiresorptive or antiangiogenic medication use nor previous radiation therapy in the area, excluding the possibilities of MRONJ and osteoradionecrosis. Nonetheless, several other causes of osteonecrosis or osteomyelitis, similarly presenting with bone necrosis and exposure, have been described (34), including DM (34-37). Regarding the latter, several mechanisms can explain the increased predisposition of diabetic patients for jaw inflammatory conditions, including altered immune responses and alterations in bone metabolism, vasculature and cell function (38,39). Considering that our patient was an older diabetic, DM-related osteomyelitis or osteonecrosis were entertained in the differential diagnosis. An additional contributing factor for our patient could be the chronic use of systemic steroids, which have been implicated in development of osteonecrosis in the presence or not of other related factors, such as antiresorptive medications (40-44). A first incisional biopsy revealed only necrotic tissue; although these findings could be interpreted as compatible with osteonecrosis, it was deemed necessary to proceed with additional tissue sampling, taking into account the rather aggressive appearance of the lesion and the possibility of biopsy sampling error. Indeed, evaluation of repeat biopsy specimen showed unequivocal evidence of DLBCL. A similar case was reported by Lee *et al*. (27): a 90 years old female patient with osteoporosis under alendronate treatment for over 10 years developed gingival edema and erythema in the posterior mandible, accompanied by a diffuse osteolytic lesion in the area. Despite the lack of exposed bone, the lesion was initially considered compatible with early stages of MRONJ, however, it was refractory to antibiotic treatment and bone debridement. The authors emphasized that early stages of MRONJ, due to nonspecific diagnostic features, can mimic malignancies as DLBCL, histopathologic examination remaining the golden standard for definitive diagnosis. Another case of oral DLBCL in a 66 years old osteoporotic woman presenting with long term mental numbness and facial swelling was reported by Zadik *et al*. (22); again, the clinical features of a nonhealing extraction site with exposed bone, along with the use of risedronate, misled to an initial diagnosis of BRONJ. Overall, the necessity of biopsy and microscopic examination when dealing with destructive jaw bone lesions cannot be overemphasized, and, on some occasions as exemplified by our case, multiple biopsies may be needed in order to obtain sufficient representative material for adequate diagnosis.

Our case also shows similarities with reported examples of other malignant tumors of primary or metastatic origin and variable histopathologic subtype mimicking osteonecrosis of the jaws (ONJ), both clinically and radiographically (45-49). Bedogni *et al*. (45) described two cases of exposed necrotic bone in patients on IV bishphosphonates, affecting the anterior maxilla and the posterior mandible, respectively; the clinical and radiographic features were consistent with BRONJ; however, histopathologic examination revealed metastatic breast cancer and medullary thyroid carcinoma, respectively. Similarly, Frei *et al*. (46) reported a case of exposed necrotic bone in the left mandible of a patient receiving IV zolendronate for metastatic prostate cancer; the lesion developed in a nonhealing postextraction socket and was initially diagnosed as BRONJ and managed first with debridement and then with curettage to no avail. Eventually, a sequestrectomy was performed, along with extraction of the adjacent teeth; histopathologic examination of the removed bone revealed a combination of necrotic bone and metastatic prostate adenocarcinoma; neverhteless, there was a long delay (more than 7 months) from the initial presentation to final diagnosis. Gander *et al*. (47) described 3 more cases of ONJ-like lesions in patients undergoing long-term treatment with bisphosphonates (corresponding to 3 out of 121 or 2.5% of patients surgically treated for BRONJ by this group); a malignant diagnosis (multiple myeloma, metastatic breast cancer and oral SCC, respectively) was rendered in all 3 cases and the authors concluded that in patients with underlying malignancy, BRONJ diagnosis should be microscopically confirmed. Mauceri *et al*. (48) also described 3 cases of oral SCC presented intraorally as exposed jaw bone mimicking BRONJ in patients receiving per os bisphosphonates, while Arduino *et al*. (49) reported a case of oral SCC arising adjacent to an area of long-term BRONJ associated with IV zolendronic acid therapy, following hematopoietic stem cell transplantation for acute myeloid leukemia. Vice versa, it should be kept in mind that BRONJ lesions may mimic malignancy, as in the case presented by Bhatt *et al*. (50) in which a patient with metastatic breast cancer on IV alendronate developed a painful mandibular lesion, which was deemed compatible with metastatic focus on the basis of the clinical and imaging features alone and was initially managed with radiation therapy with lack of response; a subsequent biopsy did not show evidence of metastatic disease and a diagnosis of BRONJ was eventually made. Likewise, Pancholi *et al*. (51) reported on a case of BRONJ mimicking oral SCC in a female patient on bisphosphonates for metastatic breast cancer, and Tocaciu *et al*. (52) described two cases of osteolytic lesions in the mandible in patients receiving low dose bisphosphonates causing significant confusion between MRONJ or SCC, before the latter diagnosis was definitively made, but with considerable delay, based on careful histopathologic examination (in one case requiring repeat biopsies and evaluation of the final surgical specimen). The aforementioned cases highlight the diagnostic challenges in discriminating between malignant disease and benign, albeit significant pathologic processes, such as osteonecrosis and osteomyelitis, when facing a case of exposed necrotic jaw bone, which is also frequently accompanied by surrounding soft tissue alterations (52).

## Conclusions

In conclusion, lymphomas of the jaws, including DLBCL, can be easily misdiagnosed, due to their nonspecific clinical and imaging features and their resemblance to other more common entities, including osteomyelitis and osteonecrosis. A high level of suspicion, along with good knowledge of the various clinical and radiographic features that the lesion may assume (such as an osteolytic lesion presenting with exposed necrotic bone and/or soft tissue ulceration), will allow to include oral NHL (and DLBCL in particular) in the working differential diagnosis. Then, adherence to the principles of diagnostic methodology, including careful review of the medical and drug history, adequate imaging, biopsy with adequate representative tissue (and repeat biopsy, if needed) and expert histopathologic analysis, will guarantee an accurate diagnosis, avoiding delays in diagnosis and adequate management, ultimate improving patients’ survival.
